# Cost-effectiveness analysis of G6PD diagnostic test for *Plasmodium vivax* radical cure in Lao PDR: An economic modelling study

**DOI:** 10.1371/journal.pone.0267193

**Published:** 2022-04-25

**Authors:** Yu Nandar Aung, Sai Thein Than Tun, Viengxay Vanisaveth, Keobouphaphone Chindavongsa, Lucy Kanya

**Affiliations:** 1 Department of Health Policy, London School of Economics and Political Science, London, United Kingdom; 2 Mahidol-Oxford Tropical Medicine Research Unit, Faculty of Tropical Medicine, Mahidol University, Bangkok, Thailand; 3 Centre for Tropical Medicine and Global Health, Nuffield Department of Medicine, University of Oxford, Oxford, United Kingdom; 4 Center for Malaria, Parasitology and Entomology, Ministry of Health, Vientiane, Lao PDR; Menzies School of Health Research, AUSTRALIA

## Abstract

**Background:**

*Plasmodium vivax* (Pv) infections were 68% of the total malaria burden in Laos in 2019. The parasite causes frequent relapses, which can be prevented by primaquine (PMQ). Testing for glucose-6-phosphate-dehydrogenase (G6PD) deficiency is recommended before giving PMQ to avoid haemolysis. Because of the risk of haemolysis in G6PD intermediate deficiencies among females, Laos uses the PMQ 14-days regimen only in G6PD normal females. Among G6PD point-of-care tests, qualitative tests cannot differentiate between G6PD normal and intermediate females. Quantitative tests are required to differentiate between G6PD normal and intermediate deficiencies. However, the quantitative test lacks the cost-effectiveness evidence necessary for decision-making for large-scale adoption. This study examined the cost-effectiveness of quantitative G6PD test, with either supervised PMQ treatment or unsupervised PMQ treatment, against the usual unsupervised PMQ 8-weeks strategy. Supervised PMQ 8-weeks strategy without G6PD testing was also compared against the unsupervised PMQ 8-weeks strategy since the former had recently been adopted in malaria high burden villages that had village malaria volunteers. A budget impact analysis was conducted to understand the incremental cost and effect needed for a nationwide scale-up of the chosen strategy.

**Methods:**

A decision tree model compared the cost-effectiveness of implementing four strategies at one health facility with an average of 14 Pv cases in one year. The strategies were unsupervised PMQ strategy, supervised PMQ strategy, G6PD test with unsupervised PMQ strategy, and G6PD test with supervised PMQ strategy. Disability Adjusted Life Years (DALYs) was the effect measure. Costs were calculated from a payer perspective, and sensitivity analyses were conducted. One Gross Domestic Product (GDP) per capita of Laos was set as the cost-effectiveness threshold. Budget impact analysis was conducted using the health facility wise Pv data in Laos in 2020.

**Findings:**

Supervised PMQ strategy was extendedly dominated by G6PD test strategies. When compared against the unsupervised PMQ strategy, both G6PD test strategies were more costly but more effective. Their Incremental Cost-Effectiveness Ratios (ICER) were 96.72US$ for the G6PD test with unsupervised PMQ strategy and 184.86US$ for the G6PD test with supervised PMQ strategy. Both ICERs were lower than one GDP per capita in Laos. Following the sensitivity analysis, low adherence for PMQ 14 days made both G6PD test strategies less cost-effective. The lower the Pv case number reported in a health facility, the higher the ICER was. In the budget impact analysis, the expected budget need was only half a million US$ when the G6PD test rollout was discriminately done depending on the Pv case number reported at the health facilities. Indiscriminate roll out of G6PD test to all health facilities was most expensive with least effect impact.

## Introduction

The World Health Organization (WHO) estimated a total of 6.4 million *Plasmodium vivax* (*Pv*) malaria infections globally in 2019 [1]. 60% of these cases were estimated to be in Asia [1]. Lao People’s Democratic Republic (Laos) reported approximately 6,600 malaria cases in 2019, among which 68% were Pv cases [2]. Pv infections are notorious for relapses because of the dormant forms of parasites, called hypnozoites, that hide in the liver [3]. A review of the previous studies on Pv radical cure found that Pv relapses can happen up to 12 months after initial infection, with a recurrence rate of up to 90% [4]. Primaquine (PMQ) is an 8-aminoquinolone drug that kills the hypnozoite forms of the Pv infection [5] and prevents relapses. However, PMQ can cause haemolysis in individuals with glucose 6 phosphate dehydrogenase (G6PD) deficiency [5]. This is an X-chromosome-linked genetic disorder where red blood cells are prone to oxidized stress leading to haemolysis when exposed to oxidizing agents such as PMQ [6, 7]. G6PD deficiency is estimated to be present in a population of 400 million across the globe [8]. In Laos, its prevalence among males is estimated at 8.1% based on G6PD genotypic data [9]. Because of primaquine’s potential haemolysis side effect, WHO recommends G6PD testing before prescribing PMQ [3]. To prescribe the 14-day primaquine regimen, the tested G6PD level should be at least 30% of its normal activity [3]. When the G6PD level is less than 30% of its normal activity, a PMQ 8 weeks regimen should be provided [3]. In Laos, a PMQ 14-day dosage regimen is recommended for males with at least 30% of normal G6PD activity and females with at least 70% of normal G6PD activity, while the PMQ 8-week dosage regime is recommended for the rest of the patient types [10]. Since G6PD deficiency is an X-chromosome linked disorder, G6PD level can be normal (G6PD enzyme level of more than 80%), or deficient (G6PD enzyme level of less than 30%), or an intermediate deficiency (G6PD enzyme level between 30% and 80%) among females whereas the G6PD level can be either normal or deficient among males [3]. Because haemolysis had been reported among females with G6PD intermediate deficiency when given PMQ [7], two different G6PD cut-offs are decided between males and females in Laos [10].

For G6PD testing, the gold standard is spectrophotometry. However, it requires intensive resources to operate [11] and hence is not suitable for low-resource settings like Laos. Two types of rapid diagnostics are available for G6PD testing in resource-limited environments: qualitative test and quantitative test [3]. Qualitative tests differentiate between G6PD normal and deficiency without indicating the G6PD activity level [12]. However, previous studies have established that qualitative tests cannot distinguish between the normal G6PD level (> 80% of normal enzyme activity) and intermediate G6PD deficiency (30%–80% of normal enzyme activity) among females [3]. In the context of Pv radical cure in Lao PDR, all female patients, regardless of their qualitative G6PD test results, receive a PMQ 8-week dosage regimen since the treatment guideline recommends using this for intermediate G6PD deficiency among females [10]. In contrast to qualitative tests, quantitative tests provide the G6PD activity level on a continuous measure and hence reliably differentiate between G6PD normal females and G6PD intermediate deficiency females [13]. It is advantageous for female Pv patients in Laos since G6PD non-deficient patients, i.e., those who have at least 70% of normal G6PD activity, would be eligible for a PMQ 14-day regimen instead of a PMQ 8-week regimen [10]. In recent years, STANDARD^™^ G6PD quantitative test has become the only point-of-care G6PD test that is currently eligible for procurement using major donors’ funding such as the Global Fund and Unitaid [14]. The test has a sensitivity of 100% and specificity of 97% to 98.6% to detect G6PD activity at the 30% threshold [13, 15]. It uses a handheld analyzer and test strips to test the G6PD and haemoglobin levels in the blood [16].

Only three cost-effectiveness studies have been done elsewhere and published for G6PD point of care tests, and none have been done for the G6PD quantitative test. At the Thai-Myanmar border, Devine et al. [17] studied the cost-effectiveness of the CareStart G6PD qualitative test [18] by comparing it with the non-testing strategy where PMQ was provided without G6PD testing. The study showed that the G6PD test strategy was cost-effective by a 75% probability at an amount much lower than the willingness to pay threshold. In Brazil, where the cost-effectiveness of two G6PD qualitative tests was modeled [19], CareStart G6PD qualitative test [18] was shown to be more cost-effective than BinaxNow G6PD qualitative test [20]. In a study conducted in Vietnam, Indonesia, Afghanistan, and Ethiopia on the sex-based treatment for Pv radical cure, G6PD qualitative tests were used with the referral services for G6PD quantitative tests. Female patients with normal G6PD results were referred to a different facility for G6PD quantitative tests and further Pv radical cure treatment with Tafenoquine [21]. The study reported that referral of female patients for G6PD quantitative tests was less cost-effective than the non-referral option where PMQ 7 days regimen was provided after the normal result from G6PD qualitative tests. However, only qualitative G6PD test was studied among males [21].

Health care in Laos is delivered mainly through the public sector [22]. The highest level of health care is at the central level under which province, district, and health center levels exist [22]. Hospitals are at the central, provincial and district level. In the malaria program, under the health center, the village is the lowest level of health care where village malaria volunteers in high to moderate malaria burden areas are trained to provide malaria testing, treatment, and referral services to higher-level facilities [23]. The private sector provides malaria services mainly through a partnership mechanism with the public sector called Public-Private-Mixed (PPM) [24]. Health care financing in Laos is largely through out-of-pocket expenditures and external aid for development [22]. For the malaria program in Laos, the Global Fund has traditionally been the biggest funding source [25]. Supported by the Global Fund and other donors such as President Malaria Initiative [26] and Bill & Melinda Gates Foundation [27], malaria testing and treatment services at all levels are offered free of charge to the population.

Laos aims to eliminate all forms of malaria infection by the end of 2030. The country has been using CareStart^™^ G6PD qualitative tests at the hospital level since 2017 [28]. However, in late 2020, these tests were removed from the Global Fund’s eligible list of products for procurement [29], and Laos was therefore no longer able to use them. Recently, in the absence of reliable G6PD tests beyond the hospital level, the malaria program in Laos has rolled out the primaquine 8 weeks regimen up to the health center level [28]. In areas where there are village malaria volunteers, primaquine compliance monitoring is done by the volunteers, albeit with several implementation challenges such as weak linkages between the health facility and village volunteers for patient referral, and the inability for patients to remain in the resident village for the full duration of PMQ course, among others [28]. With the availability of donor funding for quantitative STANDARD^™^ G6PD tests, the national malaria program in Laos is considering scaling up the rollout of these tests up to the health center level in addition to the hospital level. However, in the absence of cost-effectiveness data on the G6PD quantitative test, the rollout may likely face policy, implementation, and funding challenges in the medium and long term, especially when the donor funding phases out. To help the policymakers, program managers, and funding donors with the informed investment choices, this study examined the cost-effectiveness of the quantitative G6PD test, with either supervised PMQ treatment or unsupervised PMQ treatment, against the usual unsupervised primaquine 8 weeks strategy. Supervised PMQ 8-weeks strategy without G6PD testing was also compared against the unsupervised PMQ 8-weeks strategy since the former had recently been adopted in malaria high burden villages that had village malaria volunteers. Further analysis was made to understand the budget impact from a nationwide scale up with the chosen strategy or strategies informed by the cost-effectiveness results.

## Methods

The study examined the cost and effectiveness of implementing different Pv radical cure strategies at the health center level over a 1-year time horizon in Laos. The health center level is the focus of the study because the malaria program in Laos is planning to roll out the G6PD quantitative tests to health centers in high and moderate malaria burden areas [23]. A decision tree model was developed using input data from Laos and literature reviews. A payer perspective was adopted with the focus on either the Ministry of Health or the donor. The Consolidated Health Economic Evaluation Reporting Standards (CHEERS) guidelines have been adopted in reporting the findings of this analysis (S1 Appendix CHEERS checklist).

### Decision tree model

A decision tree model was developed in R version 3.6.3. The base case model was built to calculate the cost and effect of each of the four Pv radical cure strategies in one health center in Laos, which has a total of 14 reported Pv cases in a year. A year’s data was used because malaria stratification in Laos is planned to be revised every year, depending on which malaria control interventions need to be adjusted. The number of Pv cases was taken from the mean number of Pv cases detected among health facilities in Laos in the year 2020 [30]. Among approximately 1,300 health facilities in Laos, 10% reported Pv cases in 2020, with Pv cases ranging from 1 to 273 per year. The mean Pv number from these Pv-reported health facilities was used for base case analysis.

The four strategies compared were: (i) unsupervised PMQ strategy where PMQ 8 weeks regimen was provided to all Pv cases without G6PD testing and supervised treatment for PMQ compliance, (ii) supervised PMQ strategy where PMQ treatment compliance was supervised by village malaria volunteers, (iii) G6PD test with unsupervised PMQ strategy where G6PD quantitative test was performed to all Pv cases, and PMQ was provided without supervision for treatment compliance and (iv) G6PD test with supervised PMQ strategy where G6PD quantitative test was done for all Pv cases followed by PMQ compliance which was supervised by village malaria volunteers. These four strategies were chosen because the first strategy has traditionally been used while the second strategy has been recently adopted in Laos. The third and fourth strategies are planned for roll out in the coming years.

In the first and second strategies, a probability for Pv recurrence among patients who receive 8 weeks PMQ regimen led a patient to have either recurrence or no recurrence. In the absence of studies specific to Laos, recurrence probabilities were taken from the best available literatures [4]. Since the study’s time horizon is one year, recurrence probabilities in the model were those that were observed over the same time horizon in referenced studies. Literature that reviewed the results of clinical trials in 20 countries, where 10 studies on low dose PMQ regimen (equivalent to PMQ 14 days’ regimen of Laos) and 3 studies on high dose PMQ regimen (equivalent to PMQ 8 weeks’ regimen of Laos) reached one year of follow up, reported that 28% and 17% of Pv patients had recurrences after high dose PMQ and low dose PMQ regimen respectively at 12 months [4]. However, it should be noted that recurrence rates across the different studies had a wide variation. To cover different recurrence scenarios that could arise in areas with varying malaria burdens, which is well relatable to Laos that has localized and heterogenous malaria burden across various geographic areas [23], recurrence parameters were varied by +/- 50% in the sensitivity analysis.

For recurrent cases in first and second strategies, end nodes’ effect was scaled by DALYs for PMQ 8 weeks recurrence multiplied with the number of Pv cases expected at one health center in a year and PMQ adherence rate with or without supervision. The aim of multiplying DALYs at the end nodes with the number of Pv cases was to understand the total DALYs averted by implementing a selected Pv radical cure strategy at one health facility in one year. Further scaling of that result with the adherence rate varied the recurrence probabilities—high adherence rate lowered the recurrence probabilities and increased the total DALYs averted, while the reverse was true for low adherence rate. The same scaling methodology was applied to the effect at the end nodes for non-recurrent cases. For these, DALYs for no recurrence were multiplied with the Pv case number in a year and PMQ adherence rate.

In the absence of empirical data, the adherence rate for PMQ 8 weeks regimen was assumed at 50% of the 14 days PMQ adherence rate based on a study conducted on the Thai-Myanmar border [31]. The referenced study found that 85% of the patients who took 14 days PMQ regimen without supervision completed the course [31]. This percentage could be considered optimistic as some studies showed a lower PMQ adherence rate when unsupervised [32–34]. Such wide variation in adherence rate was tested in the sensitivity analysis. For those who had supervised PMQ treatment, the adherence rate was kept at 100%. However, with the challenges in implementing supervised PMQ treatment, as mentioned briefly in the introduction section, the adherence rate may not reach 100% even when a supervised treatment strategy is in place. Potential variations in supervised treatments’ adherence rate were thus factored in the sensitivity analysis.

For the third and fourth strategies of the decision tree model, a Pv patient was led to be male or female because G6PD deficiency prevalence is different between the male and female population. According to Laos’s national malaria program data in 2020, 67% of Pv cases were males [30]. G6PD levels below 30% of normal and 70% of normal were considered G6PD deficient among males and females, respectively, following Laos’ malaria program national treatment guideline [10]. In a study conducted in Laos, G6PD was deficient in 9% of males at 30% cut-off and 13% of females at 70% cut-off [35]. G6PD test sensitivities and specificities at 30% and 70% cut-off were applied to male and female Pv cases, respectively. Test sensitivities and specificities were referenced from a field evaluation study done on SD Biosensor G6PD quantitative test in Bangladesh [13]. G6PD true deficiency cases were provided with PMQ for 8 weeks, and the effect at the end nodes was scaled by relevant DALY, the number of Pv cases in a year, and relevant PMQ adherence rate.

When the G6PD test result was false normal, G6PD deficient patients were provided with PMQ 14 days regimen, which could lead to haemolysis. The proportion that had haemolysis among PMQ 14 days’ patients in the presence of G6PD deficiency was taken from a study conducted on the Thai-Myanmar border [36]. The base case value of 13% haemolysis probability among G6PD deficient patients treated with 14 days PMQ regimen [36] was varied by +/- 50% in sensitivity analysis to factor for the parameter variation in different settings. Referral to a higher-level health facility needed to happen when a patient had haemolysis. Based on the study conducted in southern Laos, 89.6% of people that needed medical care went to a health facility [37]. After receiving a blood transfusion at the health facility, a patient would end up having either Pv recurrence or non-recurrence. There was a 0.1 probability of death among haemolytic patients for those who did not go to the hospital, which was assumed in reference to the G6PD qualitative test cost-effectiveness study done on the Thai-Myanmar border [17]. Dead patients were considered to have zero DALY aversion, and alive patients were led to have either Pv recurrence or no Pv recurrence. For G6PD true normal cases, the PMQ 14 days regimen was provided, which resulted in either recurrence or no recurrence. The end node’s effect was scaled as in other outcomes mentioned above. When the G6DP result was false deficient, patients received the PMQ 8 weeks instead of 14 days.

**Fig 1:**
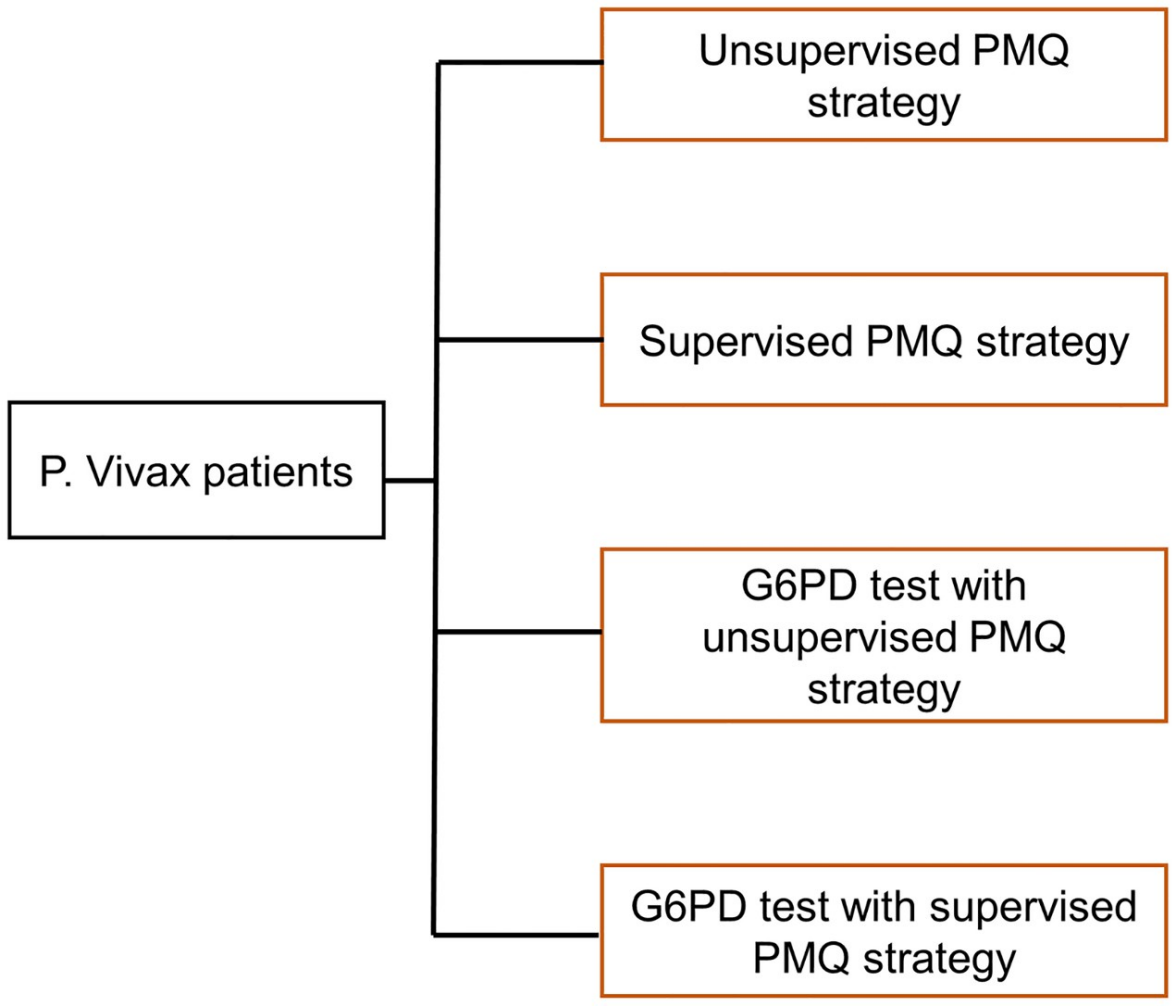
Decision tree model for four P. vivax radical cure strategies.

**Fig 2:**
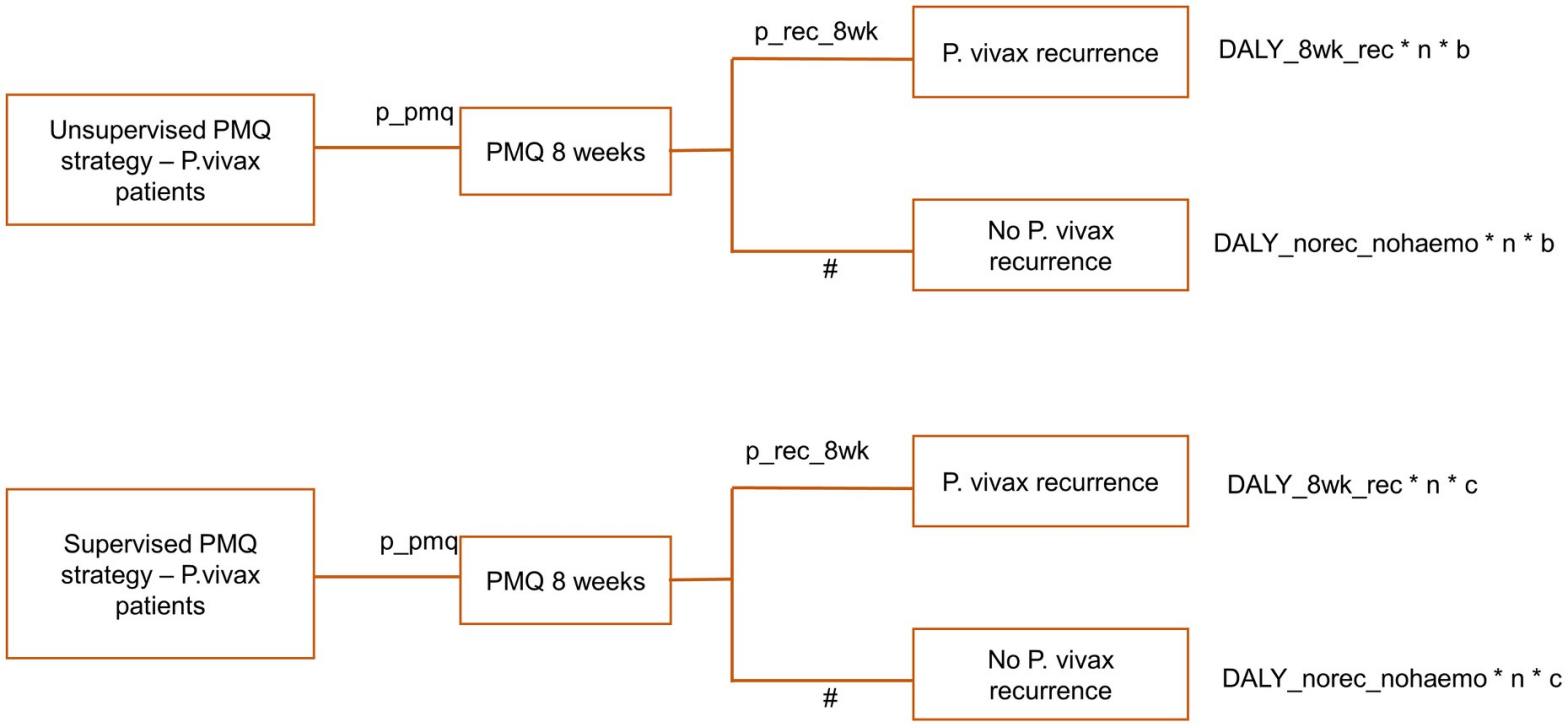
Decision tree model for unsupervised PMQ strategy and supervised PMQ strategy.

**Fig 3:**
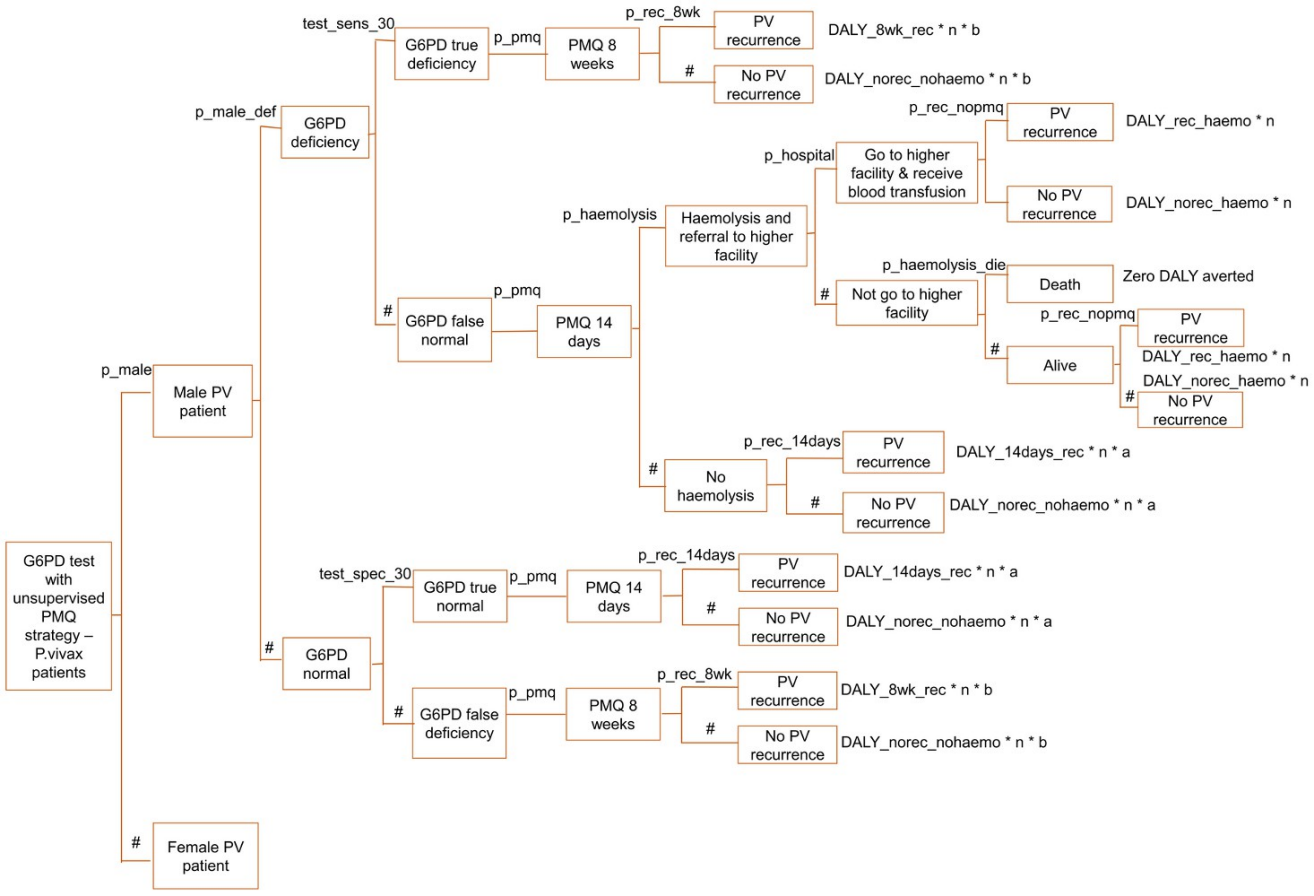
Decision tree model for G6PD test with unsupervised PMQ strategy—Male arm.

**Fig 4:**
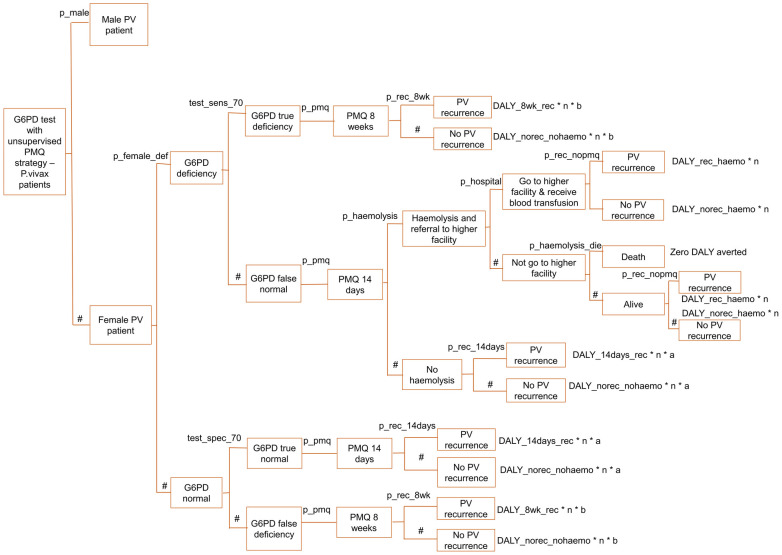
Decision tree model for G6PD test with unsupervised PMQ strategy—Female arm.

**Fig 5:**
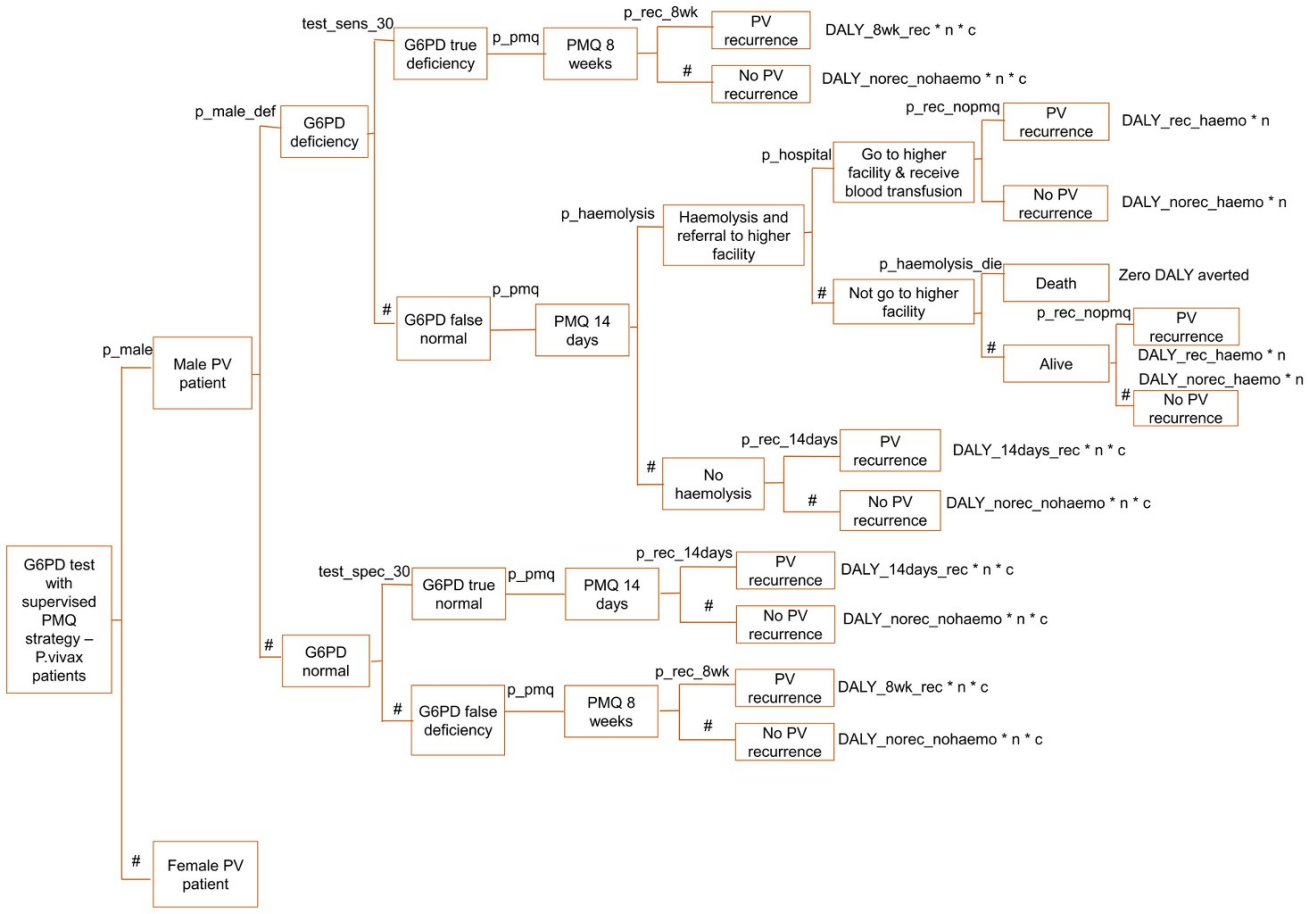
Decision tree model for G6PD test with supervised PMQ strategy—Male arm.

**Fig 6:**
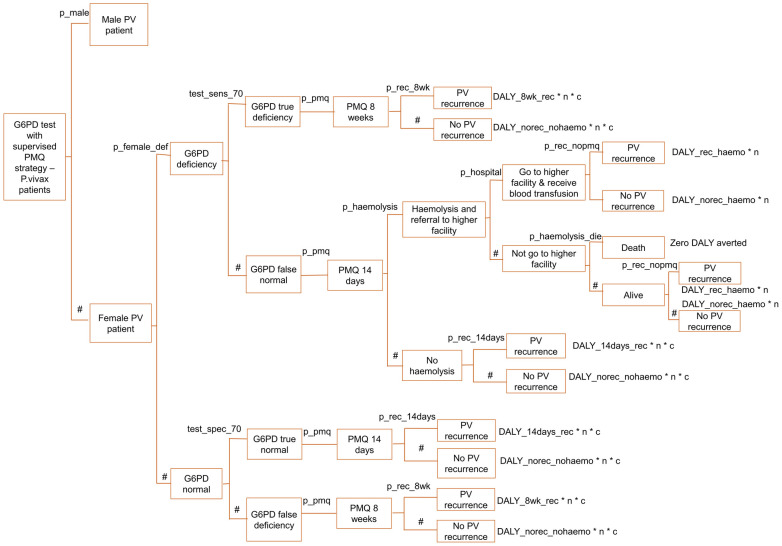
Decision tree model for G6PD test with supervised PMQ strategy—Female arm.

The decision tree model is described in Figs 1–6, and the effect measures parameters utilized in the model are presented in Table 1. Parameters related to cost inputs of the model are explained in the cost section.

**Table 1:** Parameter for effect measures included in the decision tree model.

Parameter	Symbol	Base case value	Range for sensitivity analysis		Distribution	Reference
			Low value	High value		
Mean number of PV cases at a health facility in 12 months	n	14	1	218	Gamma	[30]
Probability of receiving PMQ	p_pmq	1	not included	not included		Assumption: all P. vivax patients receive PMQ*
Proportion of PMQ 14 days patients who have P. vivax recurrence by 1 year	p_rec_14days	0.17	0.09	0.25	Beta	[4], assumed +/- 50% variation for the range
Proportion of PMQ 8 weeks patients who have P. vivax recurrence by 1 year	p_rec_8wk	0.28	0.14	0.46	Beta	[4], assumed +/- 50% variation for the range
Proportion of no PMQ patients who have recurrence by 1 year	p_rec_nopmq	0.74	0.6779	0.7923	Beta	[38]
Adherence rate for PMQ 14 days regimen without supervision	a	0.85	0.50	0.99	Beta	Base case value [31]; assumed +/-50% variation for the range***
Adherence rate for PMQ 8 weeks regimen without supervision	b	0.43	0.17	0.73	Beta	Assumption: 50% reduction from adherence rate for PMQ 14 days **
Adherence rate for PMQ regimen (both 14 days and 8 weeks regimen) with supervision	c	1	0.86	1	Beta	Base case value [31]; assumption for the range***
Proportion of male among total PV patients	p_male	0.67	not included	not included		[30]
Proportion of male PV patients who are G6PD deficient (G6PD activity less than 30% of normal)	p_male_def	0.09	0.04026	0.16829	Beta	Base case value [35]; assumption for the range***
Proportion of female PV patients who are G6PD deficient (G6PD activity less than 70% of normal)	p_female_def	0.13	0.07086	0.20364	Beta	Base case value [35]; assumption for the range***
Sensitivity of G6PD test at 30% cut off—for male PV patients	test_sens_30	1	0.887	1	Beta	[13]
Specificity of G6PD test at 30% cut off	test_spec_30	0.97	0.91	0.99	Beta	[13]
Sensitivity of G6PD test at 70% cut off—for female PV patients	test_sens_70	0.89	0.77	0.96	Beta	[13]
Specificity of G6PD test at 70% cut off—for female PV patients	test_spec_70	0.93	0.83	0.98	Beta	[13]
Probability of haemolysis among G6PD deficient patients if taken PMQ 14 days regimen	p_haemolysis	0.13	0.061	0.202	Beta	Base case value [36]; +/-50% to base case value for the range
Proportion of haemolytic patients who are referred to hospital and go to hospital	p_hospital	0.896	0.450	0.990	Beta	[37], assumed +/- 50% variation for the range
Proportion of haemolytic patients who die because of not receiving the blood transfusion	p_haemolysis_die	0.1	0.010	0.150	Beta	[17], assumed +/- 50% variation for the range
Disability weight for malaria		0.051	0.0052	0.1906	Gamma	[39]
Disability weight for haemolytic anaemia		0.149	0.1064	0.2063	Gamma	[39]
Length of illness for moderate malaria when treated		3 days	Not included	Not included		Expert opinion
Length of illness for haemolytic anaemia when treated		10 days	Not included	Not included		Expert opinion
Malaria case fatality rate in the absence of treatment		0.7	0.3	0.9	Beta	Base case value [40]; assumption for the range***
Life expectancy at age 21		51.25	42	62	Gamma	Base case value [41]; +/- 20% to base case value for the range
Median time (days) to 1st recurrence—among PMQ 14 days patients		195	80	285	Gamma	[38]
Median time (days) to 1st recurrence—among PMQ 8 weeks patients		125	110	156	Gamma	[42]
Median time (days) to 1st recurrence—among no PMQ patients		49	37	74	Gamma	[38]
PMQ dosage used in Laos	For 14 days regimen: 0.25 mg/kg/dose x 14 days >> 3.5 mg/kg					[10]
	For 8 weeks regimen: 0.75 mg/kg/dose x 8 weeks >> 6 mg/kg					

* Assumption based on the availability of PMQ stock at all health facilities in Laos.

** In the absence of empirical data, adherence for PMQ 8 weeks regimen was assumed to be 50% less than that of the PMQ 14 days regimen.

*** Ranges for these parameter values were made based on plausibility and experts’ opinions.

### Effect

The primary effect measure was Disability Adjusted Life Year (DALY) averted by each strategy, calculated using Laos’ life expectancy [41]. Disability weights for malaria and haemolytic anaemia were taken from the 2017 Global Burden of Disease Study [39]. Duration of disability in the absence of treatment was calculated by life expectancy multiplied with one minus case fatality rate [40]. Life expectancy was applied differently for five DALY categories as explained in the next paragraph. Duration of disability in the presence of treatment was assigned by expert opinion: 3 days for malaria patients without haemolysis and 10 days for malaria patients with haemolysis. Neither age weight nor discount rate was applied following the WHO’s method on global burden of disease estimates [43]. DALY averted was calculated by DALY without treatment minus DALY with treatment.

DALY was calculated for five categories: (i) DALY for no Pv recurrence in patients treated with either 14 days or 8 weeks PMQ regimen, (ii) DALY for Pv recurrence after 8 weeks of PMQ, (iii) DALY for Pv recurrence after 14 days of PMQ, (iv) DALY for no Pv recurrence in haemolytic patients, and (v) DALY for Pv recurrence in haemolytic patients. For haemolytic patients, any ongoing PMQ treatment was assumed to be withheld. There were five calculations for DALYs because the decision tree model assumed that effect and cost were measured for implementation at the health facility level. Outcomes beyond it were not considered as recurrent Pv patients may or may not come back to the same health facility for future treatment. For that reason, life expectancies for recurrent cases used the median time taken to have the first recurrent Pv episode after PMQ 8 weeks, 14 days treatment, and no PMQ. A study on the Thai-Myanmar border showed that it took 49 days to have first Pv recurrence after no PMQ and 195 days to have first Pv recurrence after 14 days PMQ regimen [38]. For median time taken to have the first recurrence among Pv 8 weeks patients, the data was referenced from a randomized trial done in Pakistan since there was no data from a setting similar to Laos [42]. Parameters for DALY calculation are presented in Table 2.

**Table 2:** Parameters for calculation of DALYs.

Parameter	No recurrence after no PMQ	Pave recurrence after 8 weeks PMQ	Pv recurrence after 14 days PMQ	No recurrence after no PMQ in haemolytic patients	Pv recurrence after no PMQ in haemolytic patients	Source
	DALY_norec_nohaemo	DALY_8wk_rec	DALY_14ds_rec	DALY_norec_haemo	DALY_rec_haemo	
Disability weight	0.051*	0.051*	0.051*	0.149**	0.149**	*Disability weight for moderate malaria [39]
						** Disability weight for haemolytic anaemia [39]
Age at onset of disability (a)	21	21	21	21	21	mean age at malaria diagnosis in Lao PDR [30]
Duration (year) of disability (without treatment)	15.375	0.10274	0.160274	15.375	0.040274	Life expectancy x (1—case fatality rate)
Duration (year) of disability (with treatment)	0.00822*	0.00822*	0.00822*	0.027397**	0.027397**	* Expert opinion: 3 days of disability with treatment
						** Expert opinion: 10 days of disability with treatment
Life expectancy at age a	51.25*	0.34246**	0.534246***	51.25*	0.13425****	* [41], ** [42], *** [38], **** [38]
Case fatality rate	0.7	0.7	0.7	0.7	0.7	[40]
DALYs averted	0.7837	0.0048	0.0078	2.2868	0.0019	

Life expectancy at age a:

** median time to first recurrence after PMQ 8 weeks was 125 days [42].

Life expectancy at age a:

*** median time to first recurrence after PMQ 14 days was 195 days [38].

Life expectancy at age a:

**** median time to first recurrence after receiving no PMQ was 49 days [38].

### Cost

Costs were calculated from the payer perspective, either the Ministry of Health, Laos, or the donors. There was no difference between these two since the Laos malaria program is funded primarily by donors except for government staff salaries and a portion of the operation cost. Once the donor funding phases out, all the costs related to the malaria program will need to be absorbed by the government. The one-year cost was calculated for one health facility with 14 Pv cases reported in 12 months. For the PMQ strategies, four cost types were included: training of health facility staff, supervision to the health facility, health facility’s human resource and operation, and PMQ cost. For the G6PD test strategies, four additional cost types were added: cost of G6PD testing, cost of G6PD biosensor, cost for G6PD quality control, and cost for treatment of haemolytic episode. For strategies that include PMQ supervision, costs related to village volunteers were added, which were training for volunteers, supervision to volunteers, incentives, and reporting support for volunteers.

All the cost incurred in Lao KIP was converted to US$ by using the Bank of Laos reference exchange rate for the year 2020 [44]. Because of the short time horizon for the study, the discount rate was not applied.

Cost data are described in Table 3.

**Table 3:** Cost input parameters, cost for one year of implementation at one health facility.

Parameter	Base case value (US$)	Range for sensitivity analysis		Distribution	Reference
		Low value (US$)	High value (US$)		
Cost of training for health facility—PMQ strategies	56.29	30.00	96.00	Gamma	[45]
Cost of training for health facility—G6PD test strategies	112.59	62.00	179.00	Gamma	[45]
Cost of supervision to health facility	188.93	99.00	307.00	Gamma	[45]
Cost of human resource and operation for health facility	538.01	276.00	922.00	Gamma	[46]
Cost of G6PD test strip—one unit	4.31	2.24	6.77	Gamma	[47]
Cost of G6PD test hand-held analyzer	147.76	77.00	245.00	Gamma	[47]
G6PD test total cost for a health facility	159.29	Linked to G6PD test strip unit cost		Gamma	[47]
G6PD test quality control cost	22.176	11	40	Gamma	[47]
Cost for PMQ 8 weeks one course	2.03	1.05	3.30	Gamma	[45]
Cost for PMQ 14 days one course	1.02	0.56	1.69	Gamma	[45]
Cost of blood transfusion for a haemolytic episode	116.07*	64	177	Gamma	[48, 49]
Cost of training for village volunteers	350.65	175.00	625.00	Gamma	[45]
Cost of supervision to village volunteers	74.97	38.00	122.00	Gamma	[45]
Incentive cost for village volunteers	149.27	75.00	265.00	Gamma	[45]
Cost of monthly reporting to health facility by village volunteers	88.63	45.00	151.00	Gamma	[45]

* cost of blood transfusion for a haemolytic episode was calculated by summation of cost for 7 days hospitalization in Lao PDR and cost for 1 unit of blood bag estimated for low and middle income countries.

#### Training cost for health facility staff

Training cost for health facility staff was taken from the Global Fund’s Regional Artemisinin-resistance Initiative 2 Elimination (RAI2E) grant, implemented in Laos from 2018 to 2020 [45]. Although G6PD quantitative tests were not introduced until 2020, training cost data were available for the G6PD qualitative test used in Laos from 2017–2020. Its cost was assumed to be the same for quantitative tests training.

#### Supervision, human resource, and operation cost for health facility

Supervision cost for a health facility was calculated for one year, using RAI2E’s 2020 cost data. Supervision cost was not linked to the number of Pv cases since supervision visits were supposed to be carried out for all health facilities regardless of the malaria burden. For human resource and operational costs for a health facility, the government’s monthly cost to support a health facility [46] was proportionated with the expected level of effort directed to malaria services. The level of effort was calculated from the expected total number of malaria tests per day divided by the health facility’s expected total average number of consultations per day, which was 20 [30]. The expected total number of malaria tests was calculated from the malaria test positivity rate of 1.8% and the proportion of Pv cases among total malaria cases in 2020, which was 68% [30]. The level of effort was capped at 80% to avoid a percentage larger than a hundred for health facilities with a very high number of Pv cases.

#### Cost related to G6PD test and PMQ

G6PD biosensor was assumed to last for three years; hence, its three-year capital cost was annuitized to obtain a year’s cost using the following formula: K was the capital cost, E was the annual cost and r was the discount rate of 3% [50].


$$K=E+\frac{E}{\left(1+r\right)}+\frac{E}{{\left(1+r\right)}^{2}}$$

For total G6PD test cost required for a year, the model was programmed to take the cost based on either of the two calculations, whichever was higher: (a) total test consumption based on reported Pv numbers in a year for a health facility or (b) minimum stock quantity required for a year based on packaging size (25 tests per box) and tests’ shelf life that remains upon arrival at health facilities (8 months). This was done because the cost of G6PD tests could not simply be attained by multiplication of G6PD test unit cost and the number of Pv cases. Other factors such as minimum stock quantity based on packaging size (e.g., if the packaging comes with 25 tests per box, a whole box of G6PD tests would need to be provided to a health facility even if it has only a few reported Pv cases) and the shelf life had to be considered. For this reason, the G6PD test minimum stock for a year was calculated by summation of 25 tests which was the number of tests that came in a box that would last for eight months (remaining shelf-life by the time the tests arrive at the health facilities) and 12 tests which were half of the number of tests in a box that can be used for the remaining four months of a year. Cost of blood draw for G6PD testing was considered to be included under the G6PD test unit cost since the test box contains all the items necessary for a finger prick test.

G6PD test quality control was assumed to be done once every three months by district or province-level staff during their supervision visits to a health facility. For the unit cost, the cost of the G6PD test quality control kits containing ten units of test was divided by 10. To get the G6PD test quality control cost needed for a health facility for a year, the unit cost was multiplied with four, which was the number of quality control tests for a health facility in a year. The cost of the G6PD test quality control kit was referenced from RAI3E grant data [47]. The frequency of G6PD test quality control was aligned with the currently practiced supervision visit frequency in Laos [45, 47]. The cost of the additional G6PD test strip used for quality control was not included in the calculation since the existing stock at the health center should be able to cover for it.

Cost for PMQ 8 weeks and 14 days courses were calculated by multiplying the unit cost for PMQ one tablet with the total number of tablets required for each course [10].

All the unit cost for PMQ, G6PD test, and device, including the international procurement and in-country supply chain cost, were taken from RAI2E [45] and RAI3E data [47].

#### Cost for haemolytic episodes

The cost for treatment of one haemolytic episode was calculated by summation of cost for seven days’ hospital stay in Laos and cost for one blood bag. Each cost referenced was from past years; hence, the Bank of Lao PDR’s annual inflation rate [51] was applied to get the adjusted cost for 2020.

#### Cost related to village volunteers

For strategies involving PMQ supervision, village volunteers’ costs were included because village volunteers supervised PMQ compliance. The cost for six village volunteers for a year was calculated since there is an average of six volunteers under one health facility in Laos [45]. As PMQ supervision was not the sole task for volunteers, total incentive and reporting support cost provided to volunteers were proportionated by the level of effort expected of the volunteers from monitoring the total number of Pv cases diagnosed at the health facility where these volunteers report to. 20% level of effort was assumed for health facilities that had 14 number of Pv cases in a year. The 20% was reached by an assumption that one volunteer will monitor approximately 2 to 3 Pv cases in a year for compliance while at the same time conducting an average of 120 malaria tests a year.

### Cost-effectiveness analysis

Incremental Cost-Effectiveness Ratio (ICER) for a base case was calculated by dividing the net cost (cost of an intervention minus cost of an alternate intervention) with net effect (DALYs averted by an intervention minus DALYs averted by an alternate intervention). The unsupervised PMQ strategy was considered the conventional strategy against which the comparison was made with other strategies. Strategies with strong dominance and extended dominance were identified. Strong dominant strategies cost more and bring less effect than all the other strategies in comparison [50]. To determine the extended dominance, interventions were ranked by cost, starting with the lowest cost intervention, and ICER was calculated for an intervention against the next lowest cost intervention. An intervention with a higher ICER in the middle of the rank, i.e., ICER higher than a lower cost intervention or a higher cost intervention, is considered extended dominance [50].

Sensitivity analysis was done by both one-way deterministic analysis and probabilistic sensitivity analysis (PSA). ICERs for all interventions regardless of extended dominance were included in one-way sensitivity analysis. For one-way sensitivity analysis, variation was made for one parameter at one time to observe how ICER was changed. Lower and upper values for one-way analysis were taken from the range determined for probabilistic sensitivity analysis. For PSA, the Monte Carlo simulation was run 10,000 times [52] by programming a random selection of parameter values from defined distributions. Gamma distribution was applied for the cost, disability weight, and Pv case number. Beta distribution was used for proportion variables. By using the formula “λ = r / point estimate of base case value” for gamma distribution and “Mode = point estimate of base case value = (α-1) / (α + β -2)” for beta distribution [53], plausible mean, median and ranges were generated which were matched against the literature if available, and if not against experts’ opinions from the national malaria program and the project data. Parameter values are presented in Tables 1 and 2. PSA results were plotted on a cost-effectiveness plane. In the absence of country specific cost-effectiveness threshold, 2019’s Gross Domestic Product (GDP) per capita from Laos, 2,534 US$ [54], was used as a cost-effectiveness threshold [55]. Using the PSA iteration results, the cost-effectiveness acceptability curve was plotted to analyze the probability of cost-effectiveness for intervention at various levels of willingness to pay [50, 56].

### Budget impact analysis

Malaria funding committed by donors and the government for Lao PDR for 2021 is approximately 8 million US$: 2 million US$ from the domestic financing [57], 5 million US$ from the Global Fund’s Regional Artemisinin resistance Initiative 3 for Elimination (RAI3E) grant [58], and around 1 million US$ from the President Malaria Initiative [59]. The amount is still 2 million short of the 10 million US$ forecasted to be required for malaria elimination in Lao PDR for 2021 [23]. Limited fiscal space from both foreign aid funding [60] and domestic funding with a declining malaria burden underscores the need for efficient allocation of resources. For policymakers, the interest would be on the budget implication of the chosen intervention/s and the potential impact that the investment brings, which will inform trade-offs within the available budget ceiling. In this analysis, the incremental budget required for a year’s implementation of the chosen intervention/s at a nationwide scale was calculated using base case parameter values. Potential DALYs averted from each investment scenario were also estimated.

In Laos, hospitals exist at the provincial and district level, whereas health centers are at the sub-district/village level. Based on these levels of health facilities and Laos’ national strategic plan [23], scenarios for incremental budget impact analysis were considered. Three potential scenarios were included: (i) scenario 1 –G6PD test with unsupervised PMQ strategy in all hospitals, but only in health centers with more than or equal to five reported Pv cases in a year, whereas, unsupervised PMQ strategy was for health centers with less than five reported Pv cases in a year; (ii) scenario 2—G6PD test with unsupervised PMQ strategy in all hospitals and health centers regardless of the Pv case number; (iii) scenario 3—G6PD test with supervised PMQ strategy in all hospitals but only in health centers with at least five reported Pv cases in a year, whereas unsupervised PMQ strategy for the rest of the health centers. The reason why the cut off was made at five Pv cases per year for the first and third scenarios was to align with the malaria national strategic plan, which will prioritize the G6PD test availability in health centers with five reported cases per year, albeit that was not specifically meant for Pv cases [23]. Besides, G6PD test availability in all hospitals has been the practice in Laos [23]. Scenario with nationwide scale-up of G6PD test with supervised PMQ strategy was excluded from the analysis because village volunteers are not present in all villages but concentrated only in moderate to high malaria burden areas, which are the areas where Pv cases are reported [45]. The results from these three scenarios were compared against the reference scenario, which was an unsupervised PMQ strategy in all hospitals and health centers. The national malaria program’s health facility-wise data on reported Pv cases in 2020 was used to calculate the budget implication and the effect on DALYs aversion. Human resource and operation costs for health facilities were proportionated with the level of effort expected by the number of reported Pv cases. In health facilities that did not report any Pv case, the level of effort was considered equivalent to that of a health facility with one Pv case per year because of the effort needed to be put in to maintain the malaria testing rate. The same approach was taken to calculate the cost of village volunteers’ incentives or the relevant reporting cost for PMQ compliance monitoring. For DALYs calculation, life expectancy related to each age group category [41] was matched against the age of the individual Pv case reported in 2020.

## Ethical approval

Ethical approval was not relevant for this modeling study. Lao PDR’s malaria program data were utilized with permission from the Center for Malaria, Parasitology, and Entomology, Ministry of Health of Lao PDR.

## Results

### Base case analysis

**Fig 7:**
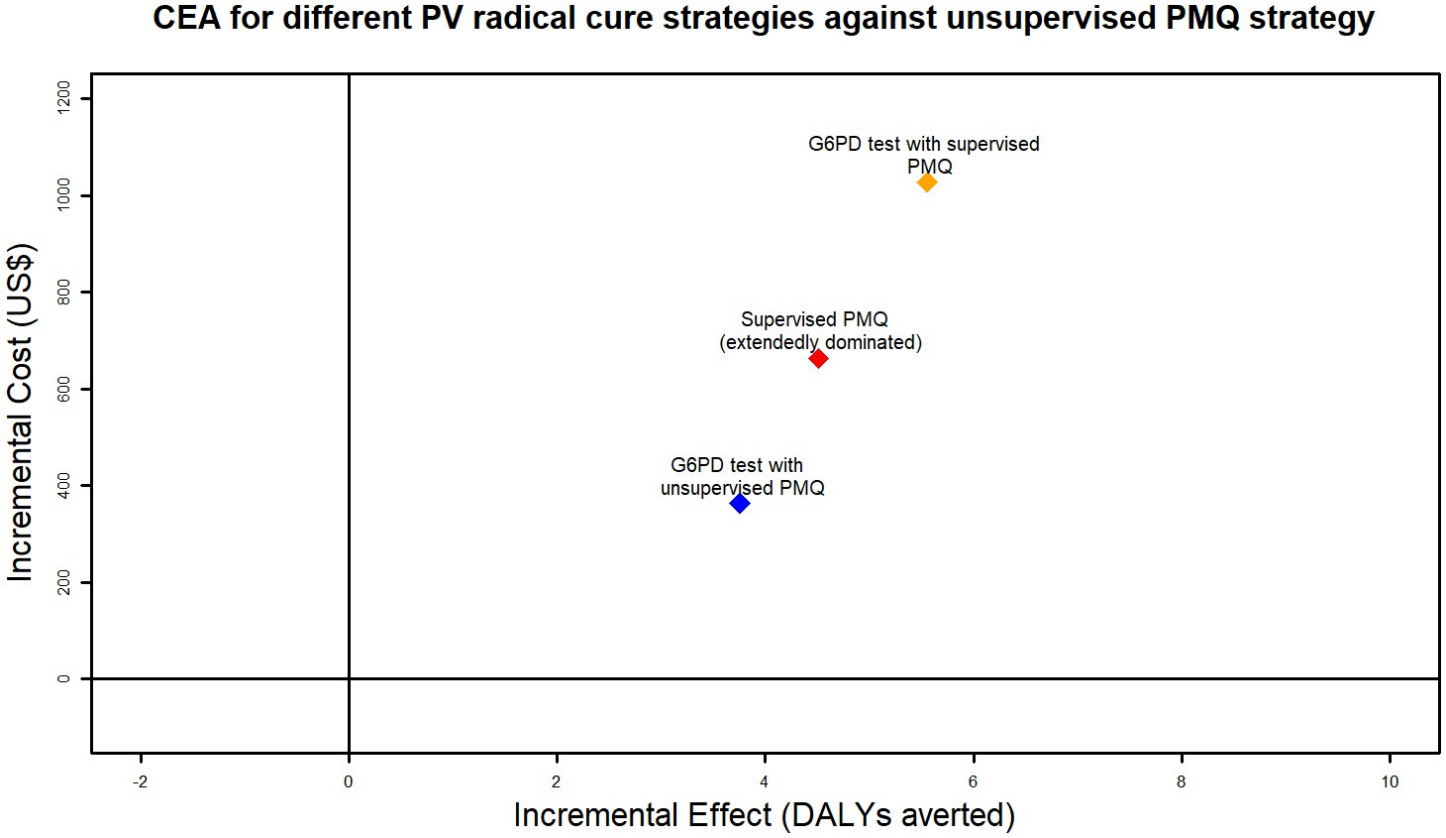
ICERs of each Pv radical cure strategy against conventional strategy (unsupervised PMQ strategy) plotted on cost-effectiveness plane.

None of the interventions included in the analysis had strong dominance, i.e., higher cost and lower effect. However, the supervised PMQ strategy was extendedly dominated by the G6PD test with unsupervised PMQ strategy and the G6PD test with supervised PMQ strategy. The ICER for the G6PD test with unsupervised PMQ strategy compared against the conventional unsupervised PMQ strategy was 96.72. This means that an extra 96.72 US$ will need to be spent for the G6PD test with unsupervised PMQ strategy to avert one additional DALY compared to the unsupervised PMQ strategy. The ICER for the G6PD test with supervised PMQ strategy against the conventional strategy was 184.86. Details of the base-case analysis are shown in Table 4, and ICERs plotted on the cost-effectiveness plane are shown in Fig 7.

**Table 4:** Cost, effect and ICER from base case analysis, where each strategy was modelled for implementation at one health facility for one year.

Intervention name	Cost (US$)	Effect (DALYs averted)	ICER when compared to lowest cost intervention
Unsupervised PMQ strategy	811.69	3.41	-
G6PD test with unsupervised PMQ strategy	1,174.98	7.16	96.72
Supervised PMQ strategy	1,475.21	7.92	Extendedly dominated
G6PD test with supervised PMQ strategy	1, 838.5	8.96	184.86

### Deterministic sensitivity analysis

One-way sensitivity analysis was done for G6PD test with unsupervised PMQ strategy versus unsupervised PMQ strategy, G6PD test with supervised PMQ strategy versus unsupervised PMQ strategy, and supervised PMQ strategy versus unsupervised PMQ strategy using the parameter values listed in Tables 1 and 3.

**Fig 8:**
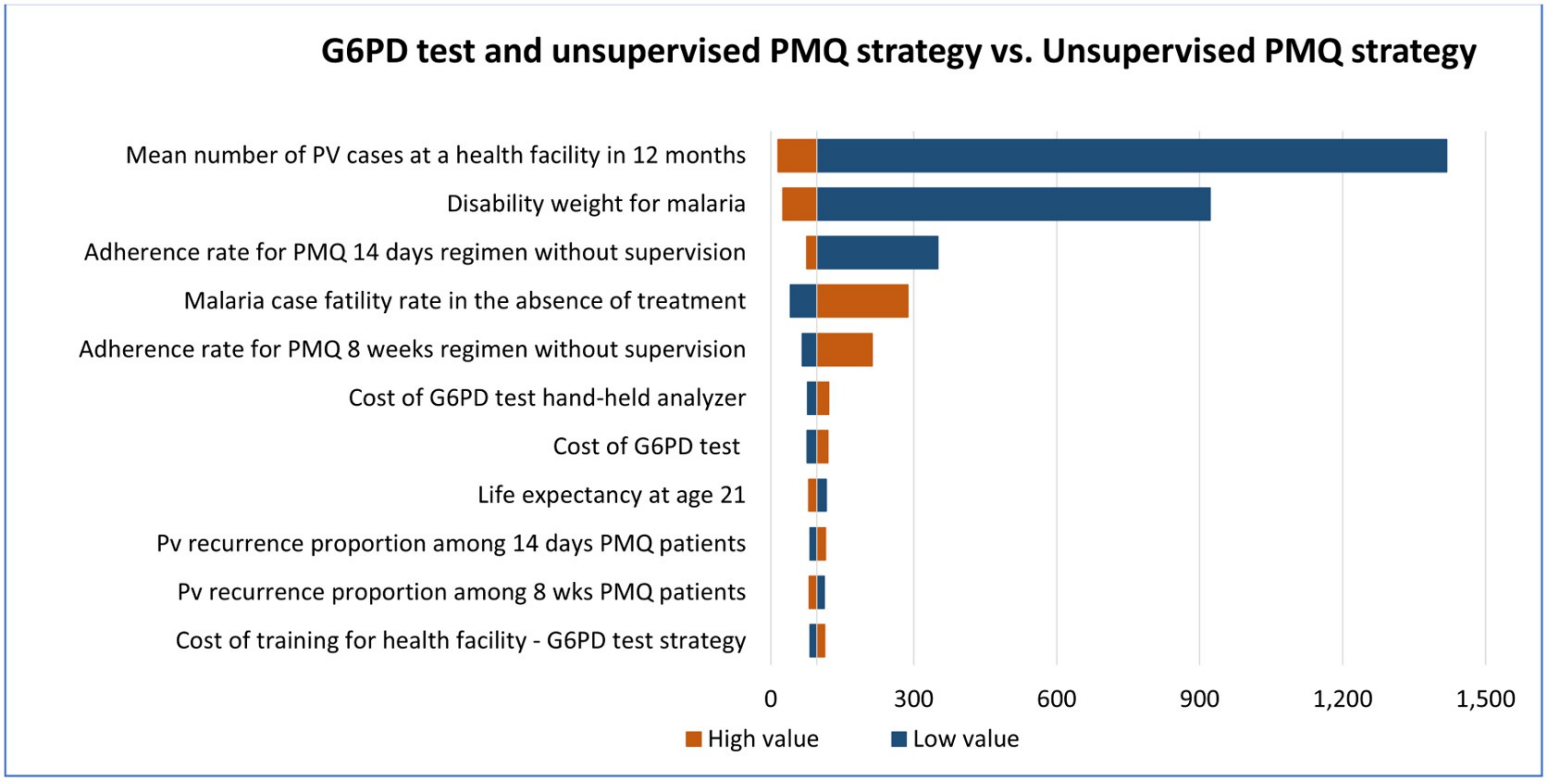
Deterministic one-way sensitivity analysis for G6PD test with unsupervised PMQ strategy against unsupervised PMQ strategy. The vertical central line represents the base case ICER.

**Fig 9:**
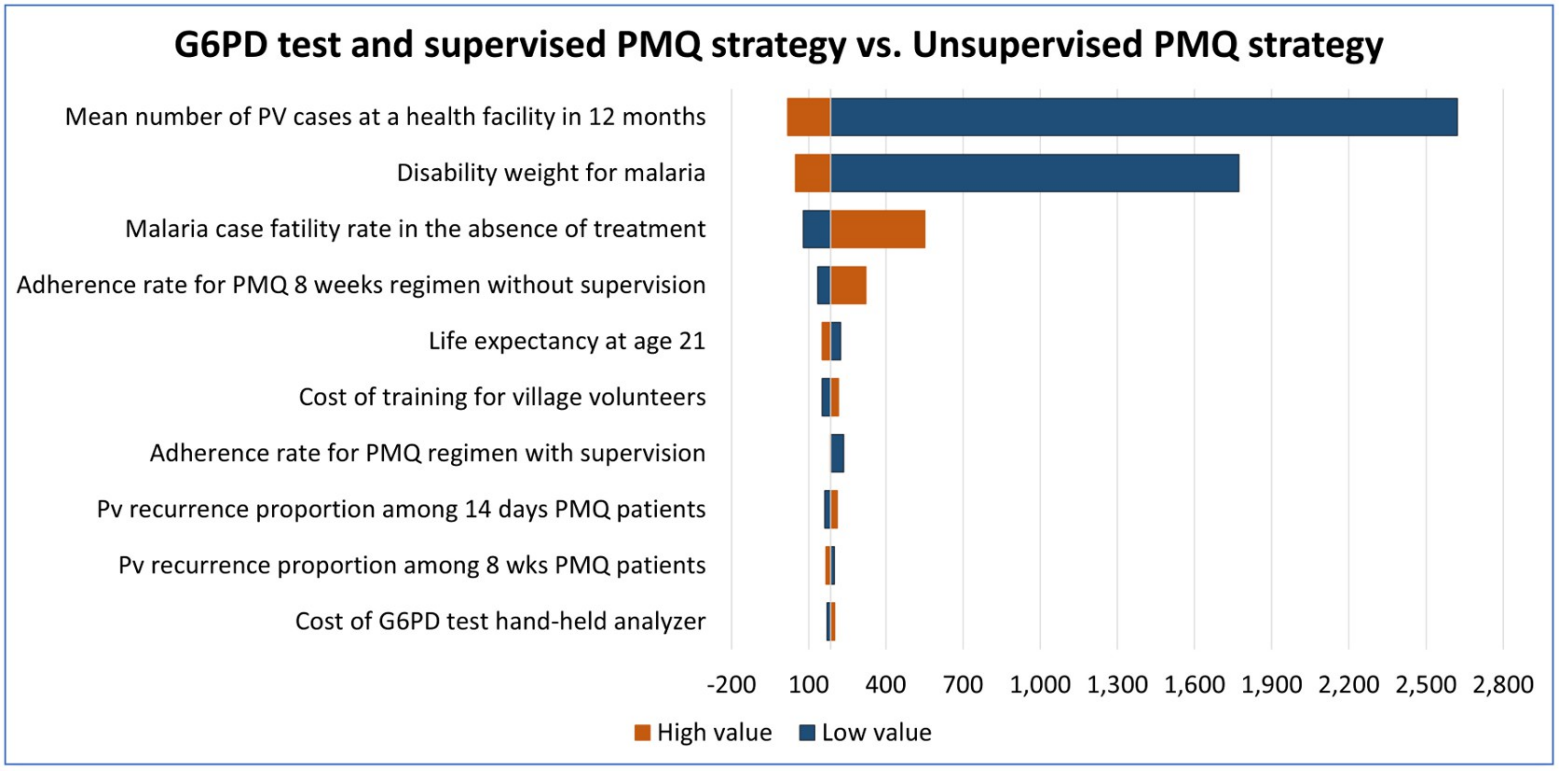
Deterministic one-way sensitivity analysis for G6PD test with supervised PMQ strategy against unsupervised PMQ strategy. The vertical central line represents the base case ICER.

**Fig 10:**
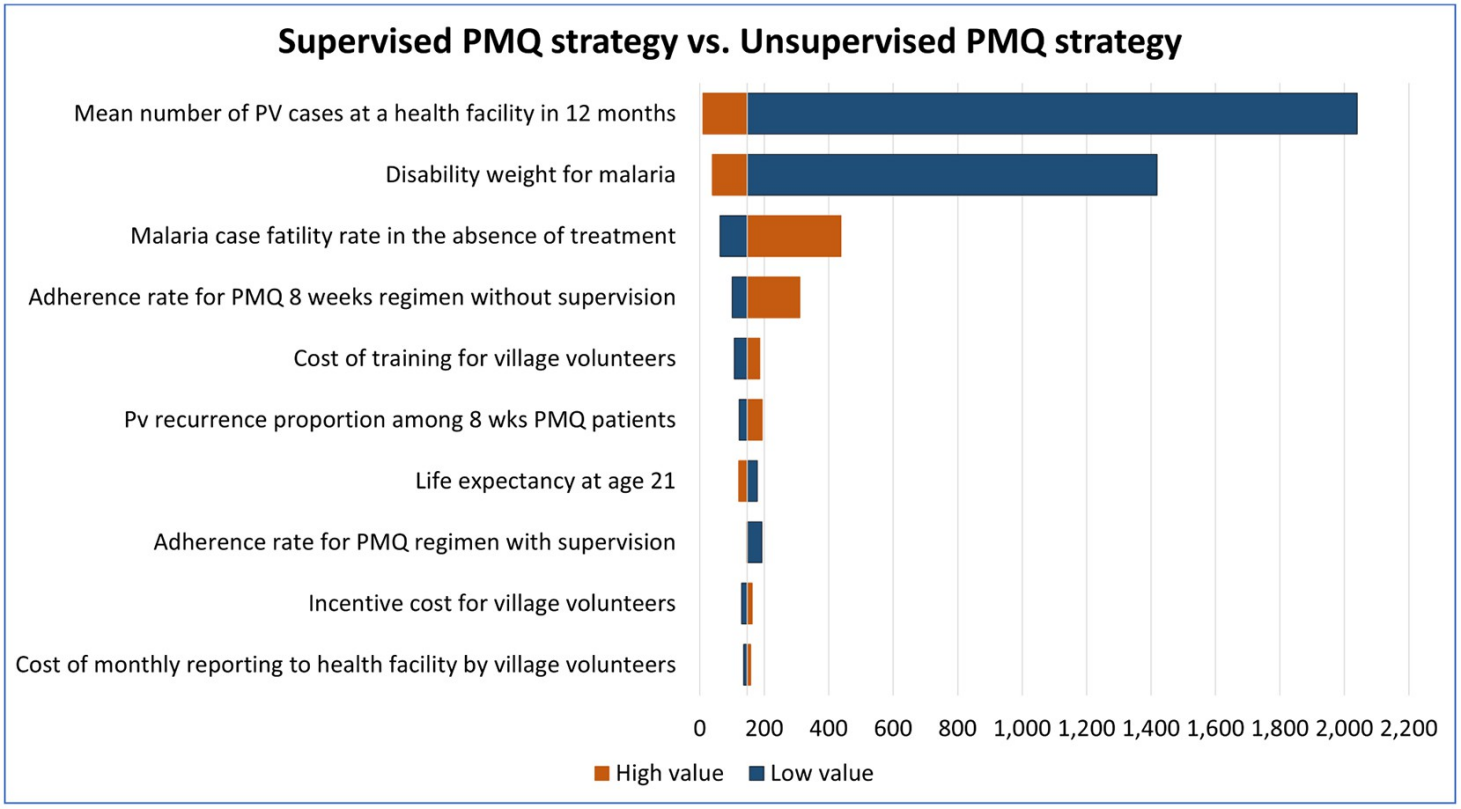
Deterministic one-way sensitivity analysis for supervised PMQ strategy against unsupervised PMQ strategy. The vertical central line represents the base case ICER.

The top 10 parameters with the biggest influence on the ICERs are shown in Figs 8–10. In all comparisons, similar results were found. Parameters with the biggest effect on ICERs were: Pv mean number reported in a health facility over 12 months, disability weight for malaria, the adherence rate for PMQ, malaria case fatality rate without treatment, and Pv recurrence rate. Lowering the mean number of reported Pv cases, lowering the disability weight for malaria (i.e., having more mild forms of malaria), or increasing the malaria case fatality rate increased the ICER, meaning the strategies of interest will be less cost-effective than the reference strategy. In all comparisons, the increased adherence rate for unsupervised PMQ 8 weeks resulted in higher ICER, i.e., all three comparator strategies became less cost-effective than the unsupervised PMQ strategy. It was because the unsupervised PMQ strategy focused only on PMQ 8 weeks regimen and averted more DALYs from the increased adherence rate for the unsupervised PMQ 8 weeks regimen while the other three comparators had none or smaller increase in DALY aversion. However, when the adherence rate for PMQ 14 days regimen or adherence rate for PMQ supervision was reduced, the ICER for G6PD test strategies was increased, i.e., G6PD test strategies became less cost-effective. Increased Pv recurrence among 14 days PMQ patients resulted in higher ICER for both G6PD test strategies and increased Pv recurrence among PMQ 8 weeks patients resulted in higher ICER for supervised PMQ strategy, meaning all three comparators became less cost-effective. Detailed results of the ICERs in each comparison with the change in each parameter are presented in the Supplementary Appendix (S2 Appendix, ICERs by one-way sensitivity analysis table).

### Probabilistic sensitivity analysis

As presented in Table 5, probabilistic sensitivity analysis resulted in an additional mean cost of 100.03 US$ (95% CI:96.55 to 103.15) to avert one additional DALY when the G6PD test with unsupervised PMQ strategy was compared against unsupervised PMQ strategy. For the G6PD test with supervised PMQ strategy, PSA reported an additional mean cost of 186.04 US$ (95% CI:180.09 to 192.43) to avert one additional DALY when compared against unsupervised PMQ strategy.

**Table 5:** Probabilistic sensitivity analysis results.

Strategy name	Mean cost—US$ (95% CI)	Mean effect—DALYs averted (95% CI)	Mean Incremental cost—US$ (95% CI)	Mean Incremental effect—DALYs averted (95% CI)	Mean ICER (95% CI)
**Unsupervised PMQ strategy**	811.52 (809.62 to 813.41)	3.44 (3.32 to 3.55)	reference	reference	reference
**G6PD test with unsupervised PMQ strategy**	1,174.53 (1,172.67 to 1,176.39)	7.07 (6.84 to 7.32)	363.01 (362.05 to 363.98)	3.63 (3.51 to 3.77)	100.03 (96.55 to 103.15)
**G6PD test with supervised PMQ strategy**	1,838.48 (1,836.40 to 1,840.56)	8.96 (8.66 to 9.26)	1,026.96 (1,025.63 to 1,028.29)	5.52 (5.33 to 5.71)	186.04 (180.09 to 192.43)

**Fig 11:**
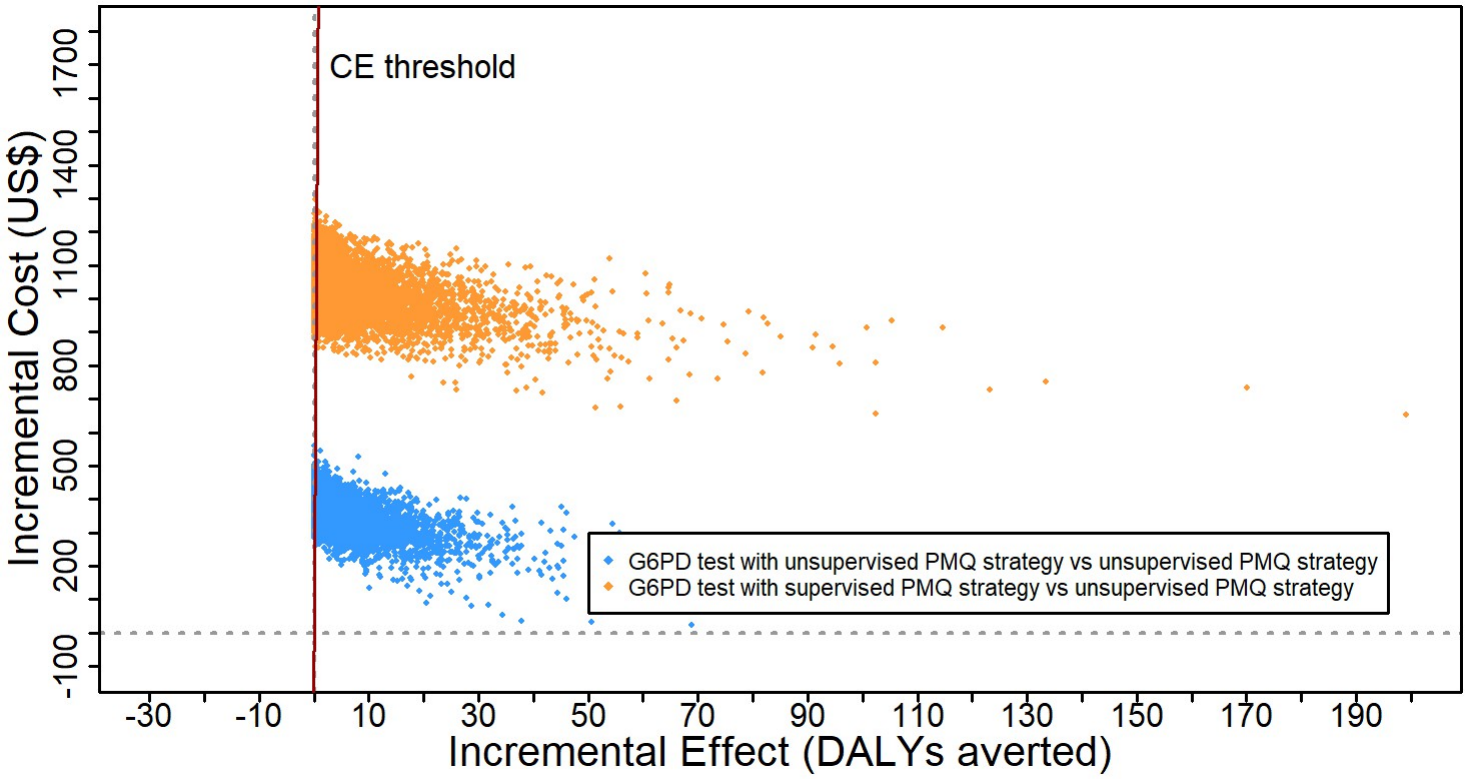
Probabilistic sensitivity analysis result for incremental cost-effectiveness of G6PD test with unsupervised PMQ strategy or G6PD test with supervised PMQ strategy, both compared against unsupervised PMQ strategy.

Fig 11 plots the 10,000 ICER iteration results on the cost-effectiveness plane and the cost-effectiveness threshold of one GDP per capita for Laos. 100% of ICERs for both G6PD test strategies against unsupervised PMQ strategy were in the north-east quadrant of the cost-effectiveness plane, meaning that both G6PD test strategies were more costly and more effective than unsupervised PMQ strategy 100% of the time. However, compared to the cost-effectiveness threshold, the G6PD test with an unsupervised PMQ strategy was cost-effective only 81% of the time. For the G6PD test with a supervised PMQ strategy, only 76% of the PSA iterations were under the willingness to pay threshold.

**Fig 12:**
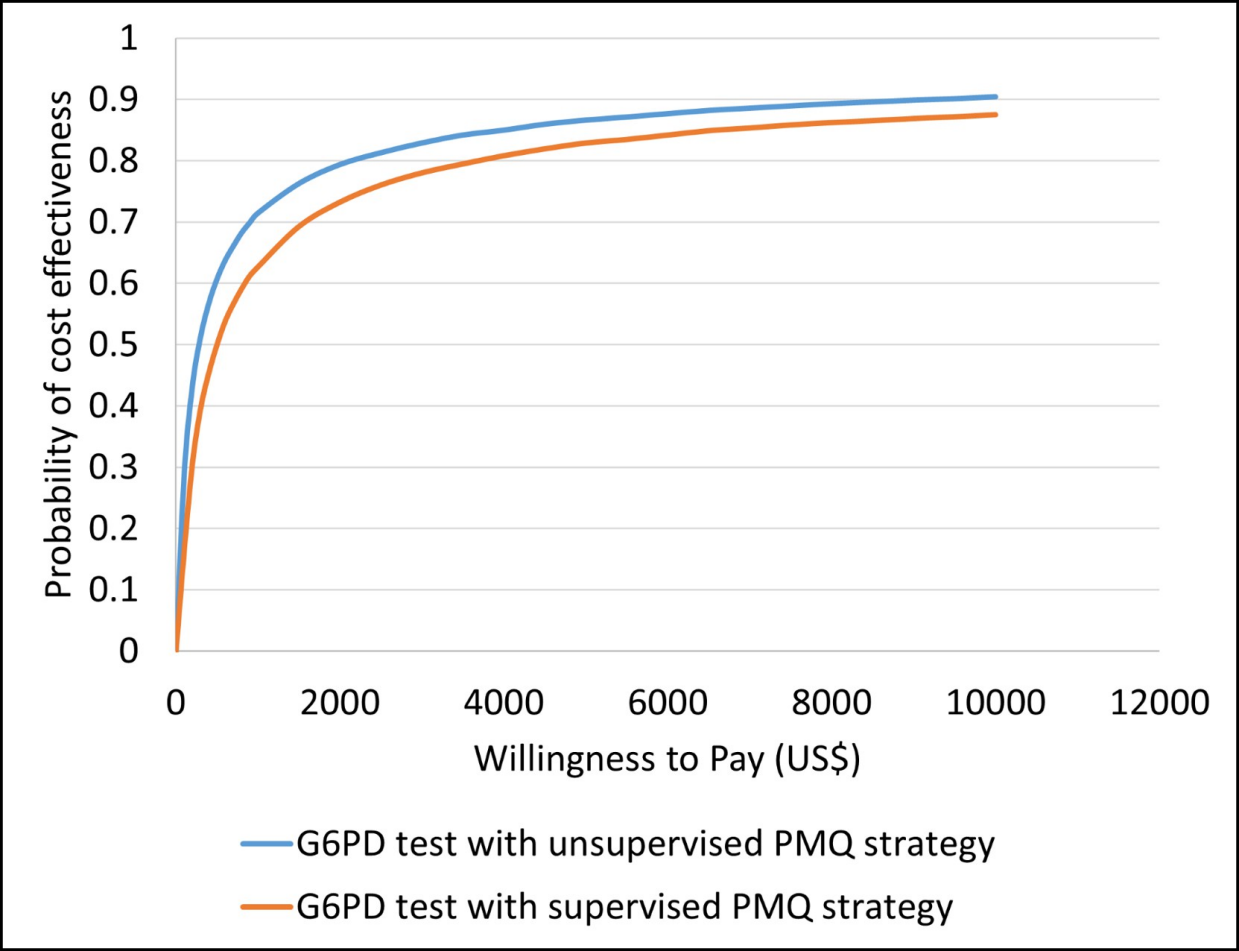
Cost-effectiveness acceptability curve for G6PD test with unsupervised PMQ strategy and G6PD test with supervised PMQ strategy, both compared against the unsupervised PMQ strategy.

Fig 12 demonstrates the cost-effectiveness acceptability curve for the G6PD test with unsupervised PMQ strategy vs. unsupervised PMQ strategy, and G6PD test with supervised PMQ strategy vs. unsupervised PMQ strategy. The curves show that the G6PD test with unsupervised PMQ strategy had a probability of 90.4% being cost-effective at a willingness to pay threshold of 10,000 US$, and the G6PD test with supervised PMQ strategy had an 87.5% probability of being cost-effective at the same threshold.

### Budget impact analysis

18% of health facilities (all hospitals and health centers with more than or equal to five Pv cases in 2020) were eligible for G6PD test strategies in scenarios 1 and 3. As presented in Table 6, results showed that all three scenarios reported a positive budget impact, i.e., higher cost than the reference scenario. G6PD test with unsupervised PMQ strategy in all health facilities (scenario 2) resulted in the highest incremental budget impact, i.e., most expensive among three scenarios. Moving away from unsupervised PMQ strategy had an additional budget requirement of 82,101 US$ for scenario 1, 203,865 US$ for scenario 3, and 445,747 US$ for scenario 2. In terms of incremental effect impact, scenario 2 reported 492.32 additional DALYs averted compared to the reference scenario. Although the incremental effect was positive and higher than that of scenario 1, which had 473.27 additional DALYs aversion, the extra cost from scenario 2 is likely to be unjustified for the policymakers. On the other hand, scenario 3 reported lower incremental budget impact, i.e., lower cost and higher incremental effect, i.e., higher DALYs aversion, compared to scenario 2.

**Table 6:** Budget impact analysis for nation-wide scale-up of different scenarios plausible to Laos’ context.

Scenario	Scenario breakdown	Cost (US$)	Total Cost (US$)	DALYs averted	Total DALYs averted	Incremental budget impact*	Incremental effect impact (DALYs averted)**
**Reference scenario**: "Unsupervised PMQ strategy" in all health facilities	-	394,392		446.30		Reference	Reference
**Alternative scenario 1**: "G6PD test with unsupervised PMQ strategy" in all hospitals and health centers with more than or equal 5 PV cases in a year and "Unsupervised PMQ strategy" in health centers with less than 5 PV cases in a year	"G6PD test with unsupervised PMQ strategy" in all hospitals and health centers with more than or equal 5 PV cases in a year	165,644	476,493	902.3	919.57	82,101	473.27
	"Unsupervised PMQ strategy" in health centers with less than 5 PV cases in a year	310,849		17.27			
**Alternative scenario 2**: "G6PD test with unsupervised PMQ strategy" in all hospitals and health centers regardless of PV case load	-	840,139		938.62		445,747	492.32
**Alternative scenario 3**: "G6PD test with supervised PMQ strategy" in all hospitals and health centers with more than or equal 5 PV cases in a year and "Unsupervised PMQ strategy" in health centers with less than 5 PV cases in a year	"G6PD test with supervised PMQ strategy" in all hospitals and health centers with more than or equal 5 PV cases in a year	287,408	598,257	1,128.89	1,146.16	203,865	699.86
	"Unsupervised PMQ strategy" in health centers with less than 5 PV cases in a year	310,849		17.27			

* Incremental budget impact was calculated by deducting the total cost of the reference scenario from the total cost of an alternative scenario.

** Incremental effect impact was calculated by deducting the total DALYs averted of the reference scenario from the total DALYs averted of an alternative scenario.

## Discussion

This study is the first of its kind in Laos and one of the few studies ever done to assess the cost-effectiveness of P. vivax radical cure strategies using the point-of-care G6PD quantitative test. The analysis is timely as Laos aims to eliminate malaria by 2030, and there is a critical need for evidence to support cost-effective Pv radical cure approaches. The results from this study will help policymakers and program managers plan and allocate resources more efficiently and strategically in eliminating a dominant form of malaria species in the country.

The results from this study point to the cost-effectiveness of both G6PD test strategies compared to the conventional approach of unsupervised PMQ strategy. However, the cost-effectiveness was sensitive to PMQ adherence and the Pv recurrence rate. In one-way sensitivity analysis, the ICER value for G6PD test strategies became larger, i.e., G6PD test strategies became less cost-effective, when the PMQ adherence rate for 14 days regimen and adherence rate for PMQ supervision was reduced from 85% to 50% and 100% to 86%, respectively. ICER values for G6PD test strategies were also increased when the Pv recurrence rate among 14 days PMQ patients was increased from 17% to 25%. The PMQ adherence rate and the Pv recurrence rate have a one-directional relationship. A low adherence rate will reduce the PMQ completion rate, hence, increase the likelihood of experiencing Pv recurrence, thus lowering the cost-effectiveness. Low adherence is expected of a drug regimen that needs to be continued even after symptoms improvement, such as PMQ for Pv radical cure. Research has shown that Pv recurrence risk could be increased by 3 to 4 folds when PMQ intake is unsupervised [61]. However, even after an investment has been made for village volunteers to supervise PMQ treatment at the village level, the implementation may come with its challenges. For example, there may not be operationally functioning referral linkages between health centers and village volunteers, or there may be different supervision strategies with different results (e.g., supervised PMQ intake every day or less frequently), or patients may not remain in the village throughout the supervised PMQ treatment, or lack of motivation by volunteers to supervise the compliance in the absence of monetary incentives specifically provided for PMQ supervision, among others. All these implementation challenges raise the question of whether shorter regimens for Pv radical cure such as PMQ 7 days or tafenoquine single dose would have better drug compliance. This study did not consider those shorter regimens because feasibility studies had not been done or planned in the near future in Laos as at the time of the analysis [23]. However, a refined cost-effectiveness analysis will be warranted once these drugs are made available to Laos.

The cost-effectiveness of the G6PD test was also largely influenced by the number of Pv cases found in a health facility over a year, which was not a surprising finding. Reduced Pv case number resulted in effect size reduction, i.e., fewer DALYs averted, and hence the intervention became less cost-effective. The reverse was true for scenarios with an increased number of Pv cases. Considering a similar level of investment made to every health facility while holding other variables constant, the only differentiating point for cost-effectiveness would be the number of Pv cases diagnosed and treated in a health facility. Therefore, a more relevant question was how the investment should be strategized, whether to roll out the G6PD test to all health facilities or only focus on a selective number of health facilities as guided by the Pv burden. The budget impact analysis of this research provided useful insights.

From the budget impact analysis, a nationwide rollout of G6PD tests to all health centers would be more costly and less effective; hence, the approach should be avoided. The choice for policymakers and program managers would be to selectively roll out the G6PD tests based on the expected burden of Pv for a health facility. Using health facility-wise Pv data in Laos from 2020, just over half a million US$ a year would be needed to have a maximum DALYs aversion from rolling out G6PD test and supervised PMQ strategy to all hospitals and health centers with at least 5 Pv cases in a year while having conventional unsupervised PMQ only strategy in the rest of the health centers. In relative terms, the amount required would be only about 10% of the Global Fund’s RAI3E grant that Laos receives from 2021–2023 [47]. For policymakers and program managers, making strategic choices does not seem remote as the country moves closer to malaria elimination. When malaria cases become very low and the funding landscape shrinks, having G6PD tests only at the hospital level likely becomes the most natural choice from a cost-effectiveness perspective.

This study has some limitations. As is the nature of the modelling studies, uncertainties with the parameter values, especially because many of them are taken from studies in other countries, can lead to variable cost-effectiveness results. Although base case analysis showed cost-effectiveness results for both G6PD test with unsupervised PMQ strategy and G6PD test with supervised PMQ strategy, the probability of each strategy being cost-effective, at probabilistic sensitivity analysis, was only 81% and 76% respectively at a cost-effectiveness threshold of 1 GDP per capita for Laos which was 2,534 US$ as at the time of this analysis. Even at a 10,000 US$ willingness-to-pay amount (close to four times GDP per capita in Laos), both G6PD strategies were cost-effective only 87–90% of the time. This was due to the wide range of parameter values in the model. When robust country-specific data is scarce, especially for PMQ adherence rate and Pv recurrence data, such a level of uncertainties will not be avoidable. On the other hand, modeling results give the policymakers an idea of how the cost-effectiveness results could turn out by different parameter values, and hence, how to improve the program implementation to achieve the favorable cost-effectiveness results. The study did not consider the societal costs, which, if included, are likely to produce smaller cost-effectiveness results [62]. Because of the limitations inherent to the decision tree models, patients were not followed after first episode of Pv recurrence. This was justified by the assumption that recurrent Pv patients after the first repeat episode may not present to the same health facility, and may go directly to a higher health facility. The study also did not include the qualitative G6DP point-of-care test because of the recent exclusion of the test from donors’ eligible list for procurement. However, even if qualitative tests become once again available for procurement, quantitative tests are likely to be the policymakers’ choice, in the long run, considering the low performance of qualitative tests among female patients [63] and a requirement for G6PD level of at least 70% by quantitative tests if tafenoquine were to be prescribed for Pv radical cure [64]. Having a field user experience with the quantitative tests will also give a competitive advantage once tafenoquine becomes available in the country. Lastly, the G6PD deficiency prevalence was obtained from a study conducted in Laos in a selected population [35], which may not represent the study population. The risk of haemolysis is also dependent on the G6PD genotype variants. A study in Laos found that the prevalence of the G6PD Viangchan variant that could cause severe haemolysis in PMQ receiving patients was up to 6% among the study population [65].

## Conclusion

G6PD test with unsupervised PMQ strategy and G6PD test with supervised PMQ strategy are found to be cost-effective at a willingness to pay threshold of 2,534 US$ when compared to unsupervised PMQ strategy. However, uncertainties in input parameters would largely influence cost-effectiveness, particularly the level of PMQ adherence and the total number of Pv cases reported by a health facility in a year. At budget impact analysis, rolling out the G6PD test to all health facilities in Laos will be the most expensive approach with fewer increments in effect. However, a more strategic rollout of G6PD tests based on the expected Pv burden will cost less with only half a million US$ in 2020, with maximum effect result. The findings from this study suggest two key policy and implementation recommendations:

1. indiscriminate roll out of G6PD test strategy in every health facility should be avoided, and instead, program managers should prioritize the roll out to a certain number of health facilities with moderate to high Pv burden.
2. once the G6PD test strategy has been rolled out, implementation challenges that could lower the PMQ adherence rate and hence the cost-effectiveness of the G6PD test should be anticipated and managed appropriately so that the strategy remains cost-effective. The choice for adding supervised PMQ treatment to improve the PMQ adherence rate would depend on the policy makers’ acceptability to incremental budget implication from such an approach. In the current context of Lao PDR, adding supervised PMQ treatment to the G6PD test strategy should be a natural thing since there are pre-existing village malaria volunteers who provide routine malaria testing and treatment services in moderate and high malaria burden villages.

## Supporting information

S1 Appendix CHEERS checklist. (PDF)

S2 Appendix ICERs by one-way sensitivity analysis table. (DOCX)
