# Supplementary material for: Cost-effectiveness analysis of G6PD diagnostic test for Plasmodium vivax radical cure in Lao PDR: An economic modelling study
Source: PLoS One. 2022 Apr 25;17(4):e0267193. doi: 10.1371/journal.pone.0267193 (PMC9037946; doi:10.1371/journal.pone.0267193)
Supplement: S2 Appendix — (DOCX) [file pone.0267193.s002.docx]

# S2 Appendix, ICERs by one-way sensitivity analysis table

**Table:** One-way sensitivity analysis, ICER results from changes to each parameter value

| **No** | **Parameter** | **G6PD test and unsupervised PMQ strategy vs. unsupervised PMQ strategy** | | **G6PD test and supervised PMQ strategy vs. unsupervised PMQ strategy** | | **Supervised PMQ strategy vs. unsupervised PMQ strategy** | |
| --- | --- | --- | --- | --- | --- | --- | --- |
|  |  | **ICER when parameter's low value was used** | **ICER when parameter's high value was used** | **ICER when parameter's low value was used** | **ICER when parameter's high value was used** | **ICER when parameter's low value was used** | **ICER when parameter's high value was used** |
| 1 | Mean number of PV cases at a health facility in 12 months | 1,418.33 | 14.65 | 2,621.57 | 17.55 | 2,040.16 | 9.36 |
| 2 | Pv recurrence proportion among 14 days PMQ patients | 82.05 | 115.21 | 161.86 | 211.95 | Not included | Not included |
| 3 | Pv recurrence proportion among 8 wks PMQ patients | 112.46 | 80.55 | 199.22 | 166.70 | 122.17 | 193.75 |
| 4 | Pv recurrence proportion among no PMQ patients | 95.82 | 95.87 | 183.51 | 183.59 | Not included | Not included |
| 5 | Adherence rate for PMQ 14 days regimen without supervision | 350.81 | 75.47 | 183.55 | 183.55 | Not included | Not included |
| 6 | Adherence rate for PMQ 8 weeks regimen without supervision | 65.66 | 212.99 | 134.87 | 323.01 | 100.95 | 310.34 |
| 7 | Adherence rate for PMQ regimen with supervision | Not included | Not included | 236.57 | 183.55 | 192.63 | 145.73 |
| 8 | Proportion of male PV patients who are G6PD deficient (G6PD activity less than 30% of normal) | 92.51 | 101.69 | 182.37 | 185.49 | Not included | Not included |
| 9 | Proportion of female PV patients who are G6PD deficient (G6PD activity less than 70% of normal) | 94.13 | 98.09 | 182.93 | 184.34 | Not included | Not included |
| 10 | Sensitivity of G6PD test at 30% cut off - for male PV patients | 95.10 | 95.85 | 183.32 | 183.55 | Not included | Not included |
| 11 | Specificity of G6PD test at 30% cut off - for male PV patients | 99.94 | 94.56 | 184.92 | 183.11 | Not included | Not included |
| 12 | Sensitivity of G6PD test at 70% cut off - for female PV patients | 95.28 | 96.18 | 183.38 | 183.65 | Not included | Not included |
| 13 | Specificity of G6PD test at 70% cut off - for female PV patients | 99.10 | 94.30 | 184.67 | 183.00 | Not included | Not included |
| 14 | Probability of haemolysis among G6PD deficient patients if taken PMQ 14 days regimen | 95.84 | 95.85 | 183.54 | 183.57 | Not included | Not included |
| 15 | Proportion of haemolytic patients who are referred to hospital and go to hospital | 95.84 | 95.85 | 183.55 | 183.55 | Not included | Not included |
| 16 | Proportion of haemolytic patients who die because of not receiving the blood transfusion | 95.84 | 95.85 | 183.55 | 183.55 | Not included | Not included |
| 17 | Disability weight for malaria | 922.26 | 25.67 | 1,772.81 | 49.14 | 1,418.59 | 38.99 |
| 18 | Disability weight for haemolytic anaemia | 95.88 | 95.85 | 183.60 | 183.49 | Not included | Not included |
| 19 | Malaria case fatility rate in the absence of treatment | 41.06 | 287.87 | 78.64 | 551.33 | 62.43 | 437.83 |
| 20 | Life expectancy at age 21 | 96.06 | 95.68 | 223.91 | 151.76 | 177.75 | 120.50 |
| 21 | Median time (days) to 1st recurrence - among PMQ 14 days patients | 95.82 | 95.89 | 183.88 | 183.30 | Not included | Not included |
| 22 | Median time (days) to 1st recurrence - among PMQ 8 weeks patients | 95.82 | 95.89 | 183.53 | 183.60 | 145.77 | 145.63 |
| 23 | Median time (days) to 1st recurrence - among no PMQ patients | 95.85 | 95.85 | 183.55 | 183.55 | Not included | Not included |
| 24 | Cost of training for health facility - PMQ strategy | 102.78 | 85.38 | 188.25 | 176.46 | 145.73 | 145.73 |
| 25 | Cost of training for health facility - G6PD test strategy | 82.50 | 113.37 | 174.51 | 195.43 | Not included | Not included |
| 26 | Cost of supervision to health facility | 95.85 | 95.85 | 183.55 | 183.55 | 145.73 | 145.73 |
| 27 | Cost of human resource and operation for health facility | 95.85 | 95.85 | 183.55 | 183.55 | 145.73 | 145.73 |
| 28 | Cost of G6PD test | 75.69 | 119.91 | 169.89 | 199.86 | Not included | Not included |
| 29 | Cost of G6PD test hand-held analyzer | 77.18 | 121.50 | 170.90 | 200.94 | Not included | Not included |
| 30 | G6PD test quality control cost | 92.90 | 100.55 | 181.56 | 186.74 | Not included | Not included |
| 31 | Cost for PMQ 8 weeks one course | 99.45 | 91.19 | 186.00 | 180.40 | 145.73 | 145.73 |
| 32 | Cost for PMQ 14 days one course | 95.14 | 96.89 | 183.07 | 184.26 | Not included | Not included |
| 33 | Blood transfusion cost for haemolytic episode | 95.84 | 95.85 | 183.55 | 183.56 | Not included | Not included |
| 34 | Cost of training for village volunteers | Not included | Not included | 152.15 | 215.97 | 107.15 | 185.55 |
| 35 | Cost of supervision to village volunteers | Not included | Not included | 176.94 | 190.17 | 137.61 | 153.86 |
| 36 | Incentive cost for village volunteers | Not included | Not included | 170.21 | 196.90 | 129.33 | 162.12 |
| 37 | Cost of monthly reporting to health facility by village volunteers | Not included | Not included | 175.75 | 193.27 | 136.14 | 157.67 |
